# Death of Sir Astley Cooper

**Published:** 1841-04

**Authors:** 


### DEATH OF SIR ASTLEY COOPER.

A London correspondent of a New York paper, under date of March 3d, mentions the death of Sir Astley Cooper, which occurred on the 12th of February. A more lengthy notice of him, and his works, may be expected shortly.

